# 非可逆偶氮共价有机骨架用于水中2，5-二氯-1，4-苯醌的吸附

**DOI:** 10.3724/SP.J.1123.2026.01012

**Published:** 2026-10-08

**Authors:** Xuqin RAN, Xu YANG, Chaoyi LONG, Hailong QIAN, Xiuping YAN

**Affiliations:** 1. 江南大学食品科学与资源挖掘全国重点实验室，江苏 无锡 214122; 1. State Key Laboratory of Food Science and Resources，Jiangnan University，Wuxi 214122，China; 2. 江南大学食品学院分析食品安全学研究所，江苏 无锡 214122; 2. Institute of Analytical Food Safety，School of Food Science and Technology，Jiangnan University，Wuxi 214122，China

**Keywords:** 非可逆共价有机骨架, 氯代苯醌, 吸附, 去除, irreversible covalent organic framework, chlorinated benzoquinone, adsorption, removal

## Abstract

2，5-二氯-1，4-苯醌（2，5-DCBQ）是一种典型的卤代消毒副产物，其高效吸附去除对于保证水资源安全具有重要意义。本文以1，3，5-三醛基间苯三酚（Tp）为醛基单体，对二氨基偶氮苯（Azo）为氨基单体，通过互变异构反应制备了一种非可逆酮烯胺式偶氮共价有机骨架（Tp-Azo），并用于高效吸附去除水中的2，5-DCBQ。所制备的Tp-Azo比表面积为824.5 m^2^/g，孔径分布约为2.0 nm。在电子给体-受体相互作用、*π*-*π*相互作用和疏水相互作用等多重机制协同下，Tp-Azo能在20 min内实现对2，5-DCBQ的快速吸附平衡，最大吸附容量高达427.4 mg/g。与已报道的去除消毒副产物的工作相比，基于Tp-Azo的2，5-DCBQ去除表现出更高的吸附容量以及更短的吸附平衡时间。在实际水体（包括自来水和泳池水）中，当2，5-DCBQ含量分别为5 mg/L和50 mg/L时，Tp-Azo对其去除效率可达87.9%~96.6%。本研究为环境体系中卤代消毒副产物的高效去除提供了新选择。

## Abstract (en)

The disinfection of water is a fundamental and indispensable process in modern public health infrastructure. It plays a pivotal role in safeguarding drinking water safety by inactivating pathogenic microorganisms such as bacteria and viruses， thereby preventing waterborne disease outbreaks. However， a significant and well-documented drawback of chemical disinfection， particularly with chlorine-based agents， is the formation of disinfection byproducts （DBPs）. These DBPs are generated through complex reactions between disinfectants and natural organic matter present in source water. Among the vast array of identified DBPs， halogenated compounds are of particular concern due to their enhanced toxicity and persistence. 2，5-Dichloro-1，4-benzoquinone （2，5-DCBQ） is a prominent representative of these toxic halogenated DBPs. Toxicological studies have conclusively demonstrated that 2，5-DCBQ can induce pronounced oxidative stress in mammalian cells. This leads to a cascade of detrimental effects including a marked increase in intracellular reactive oxygen species. Subsequently， the detrimental effects may cause DNA single-strand breaks， oxidative base damage， and chromosomal aberrations. Consequently， developing effective technologies for the targeted removal of 2，5-DCBQ from aqueous environments is of paramount importance for mitigating its associated health risks. Current removal strategies， including adsorption using solid-phase materials， often suffer from several limitations. These include reliance on a single interaction mechanism， modest adsorption capacities， or slow kinetics. This hinders their practical application for efficient 2，5-DCBQ control. To address these challenges， this study presents the design， synthesis， and application of a novel porous organic adsorbent. Termed Tp-Azo， this adsorbent is specifically engineered for the rapid and efficient removal of 2，5-DCBQ. Tp-Azo was synthesized via a condensation reaction and possesses an exceptionally high specific surface area of 824.5 m^2^/g. It incorporates a high density of electron-rich azo （-N=N-） functional groups within its framework. These groups act as strong electron donors， enabling potent electron donor-acceptor interactions with the electron-deficient quinone ring of 2，5-DCBQ. Furthermore， the predominantly aromatic backbone of Tp-Azo facilitates strong *π-π* stacking interactions with the aromatic structure of 2，5-DCBQ. The inherent hydrophobicity of Tp-Azo promotes additional adsorption via hydrophobic effects. Critically， the material possesses an average pore diameter of approximately 2.0 nm. It is substantially larger than the estimated molecular kinetic diameter of 2，5-DCBQ. This suitable pore size ensures minimal diffusion resistance， allowing 2，5-DCBQ to rapidly access the internal adsorption sites.The adsorption performance of Tp-Azo was systematically evaluated under various conditions. Under pH 2-3， the adsorption efficiency was reduced. This is attributed to the protonation of 2，5-DCBQ， which diminishes its hydrophobicity. The shielding effect of high H⁺ concentration can also weaken electrostatic interactions. In contrast， within pH 4-6， Tp-Azo exhibited consistently high removal rates. Therefore， pH 5 was selected as the optimal condition for subsequent experiments to simulate typical water treatment scenarios. The adsorption kinetics were remarkably fast within the first 3 min and complete equilibrium was reached within 20 min. The kinetics were best described by the pseudo-second-order model， indicating that the adsorption process is primarily governed by chemisorption. Isotherm studies revealed that the adsorption capacity increased with the initial 2，5-DCBQ concentration. They were accurately modeled by the Langmuir isotherm， suggesting a homogeneous adsorption surface and monolayer coverage. The calculated maximum adsorption capacity was significantly higher than that reported for many conventional adsorbents.Swimming pool water and tap water were spiked with 2，5-DCBQ at 5 mg/L and 50 mg/L. Impressively， Tp-Azo achieved removal efficiencies of 96.2% and 96.6% at 5 mg/L， 87.9% and 88.7% at 50 mg/L， respectively. Given that realistic environmental concentrations of halogenated DBPs like 2，5-DCBQ are typically in the μg/L range， this suggests that Tp-Azo is highly capable of achieving virtually complete removal in real-world applications. The study provides a novel and highly effective material enabling targeted removal of halogenated DBPs. It also offers a strategic design principle for developing advanced adsorbents for DBPs in water treatment.

水消毒处理在确保饮用水安全方面发挥着至关重要的作用^［1］^。有效的消毒可以杀灭水中的细菌和病毒，防止病原体在流动水中的传播。然而在消毒过程中，一些天然有机物与氯和氯胺等消毒剂反应时，会产生一系列对人体有潜在风险的物质，即消毒副产物（DBPs）^［2，3］^。DBPs是饮用水卫生安全重点检测对象，我国最新修订颁布的《生活饮用水卫生标准》（GB5749-2022）、世界卫生组织和美国环境保护局已对部分DBPs制定了限值规定^［4，5］^。然而，DBPs研究还处于起步阶段，一些新出现的卤代DBPs尚未被纳入监管体系，对生态环境和人体健康仍构成显著的安全隐患。2，5-二氯-1，4-苯醌（2，5-DCBQ）是一种高毒性卤代DBP，已被证实可诱导哺乳动物细胞产生显著的氧化应激反应，导致活性氧水平升高，进而引发DNA单链断裂、碱基氧化损伤和染色体畸变^［6-10］^。因此，制备高效的吸附剂用于复杂体系中2，5-DCBQ的去除，对于2，5-DCBQ的控制尤为重要。

目前，用于2，5-DCBQ去除的固相吸附剂主要是商品化的聚二甲基硅氧烷、聚二乙烯苯及十八烷基键合硅胶等，这些吸附剂通常依赖单一作用机制，吸附性能有待提高^［11-13］^。共价有机骨架（COF）作为一种结构高度有序、功能可设计的多孔有机聚合物，在污染物选择性吸附方面具有显著优势^［14-17］^。由于氯原子的强吸电子效应，2，5-DCBQ呈现缺电子特性，而偶氮（-N=N-）作为一种富电子基团，可作为电子供体与2，5-DCBQ发生强相互作用。因此制备基于-N=N-的COF不仅可以通过电子供体-受体相互作用提高2，5-DCBQ的吸附性能，其高度有序的结构还可促进2，5-DCBQ在材料内部的传质扩散，为实际样品中氯代苯醌类化合物的控制提供新的思路。

本研究以1，3，5-三醛基间苯三酚（Tp）为醛基单体，对二氨基偶氮苯（Azo）为氨基单体，在*N，N*-二甲基乙酰胺（DMAC）-邻二氯苯（1:1，体积比）体系中，通过互变异构反应制备了非可逆的酮烯胺COF（Tp-Azo）。非可逆Tp-Azo的高稳定性能有效保证了复杂环境体系中2，5-DCBQ的吸附。研究进一步考察了不同pH值下Tp-Azo对2，5-DCBQ的吸附性能，并深入探究了其吸附动力学与热力学行为，最后评估了Tp-Azo对实际环境水样中2，5-DCBQ的吸附去除性能。本研究为DBPs的污染控制提供了一种新的可行策略。

## 1 实验部分

### 1.1 仪器与试剂

电子天平（BSA124S，Sartorius，德国）；粉末X射线衍射仪（PXRD，D2 PHASER，Bruker，德国）；N_2_吸附分析仪（Autosorb-IQ，Quantachrome，美国）；傅里叶变换红外光谱仪（FTIR，Nicolet IR IS10，Nicolet，美国）；紫外可见近红外光谱仪（UV-3600PLUS，Shimadzu，日本）。

2，5-DCBQ（纯度≥98%）购于上海迈瑞尔生化科技有限公司；Tp和Azo分别购于吉林研伸科技有限公司和中国上海楷树化学科技有限公司；DMAC（≥99%）、邻二氯苯（≥99%）、四氢呋喃（≥99%）、盐酸（37%）、氢氧化钠（≥96%）和乙腈（≥99.9%）均购于上海国药集团化学试剂有限公司；超纯水购于杭州娃哈哈集团有限公司。

### 1.2 标准溶液配制

称取40.0 mg 2，5-DCBQ标准品于2 mL棕色瓶中，使用移液管量取1 mL乙腈溶解标准品，进一步用水配制成40 g/L的标准储备液，置于-4 ℃冰箱备用。在进行实验前，使用棕色容量瓶将标准品用水稀释到实验浓度，现配现用。

### 1.3 Tp-Azo的制备

采用溶剂热法制备Tp-Azo^［18］^。准确称取0.3 mmol Tp、0.45 mmol Azo于Schlenk反应管中，加入3 mL DMAC和3 mL邻二氯苯，超声10 min使单体充分分散。将Schlenk管在液氮中充分冷冻，经过脱气-充氮气3次循环，在氮气环境下封闭Schlenk管，待Schlenk管接近室温后，置于120 ℃油浴中，静置反应3天。使用四氢呋喃清洗红色材料至离心后上清液无色，在60 ℃真空干燥箱中干燥备用。产率为81%。

### 1.4 Tp-Azo吸附性能考察

#### 1.4.1 标准曲线建立

以2，5-DCBQ标准品为溶质，使用水溶解，于容量瓶中定容，配制质量浓度依次为0.125、0.25、0.5、1、5、10、25、50、100 mg/L的标准工作溶液。移取0.8 mL标准工作溶液，加入到样品池，纯净水作为空白，记录UV-Vis吸收光谱272 nm的吸收值，以标准品浓度为横坐标，吸收值为纵坐标绘制标准曲线，标准品现配现用。

#### 1.4.2 动力学吸附性能

取多个棕色玻璃瓶，各加入1 mg Tp-Azo备用。在瓶中分别加入1 mL配制好的200 mg/L 2，5-DCBQ标准溶液（pH 5），超声分散后放置于摇床上，吸附时间分别设定为45 s，以及1.5、2、3、5、10、15、20、30、45、60、90、120、180 min。离心取100 μL上清液，用纯水定容至2 mL，过膜后使用紫外可见近红外光谱仪进行定量分析，所有定量实验如无特殊说明，均进行3次平行测定。

进一步对实验数据进行伪一级动力学和伪二级动力学线性拟合，伪一级和伪二级动力学拟合公式分别见式（1）和式（2）：


$$ln\;\left(q_{e}-q_{t}\right)=ln\;q_{e}-K_{1}t$$ （1）

$$\frac{t}{q_{t}}=\frac{1}{K_{2}{q_{e}}^{2}}+\frac{1}{q_{e}}t$$ （2）

其中，*q_t_* （mg/g）为吸附时间*t*时对应的吸附量，*q*_e_（mg/g）为平衡吸附量。*K*_1_（g/（mg∙min））和*K*_2_（g/（mg∙min））分别为伪一级动力学和伪二级动力学常数。

#### 1.4.3 等温吸附性能

使用容量瓶准确配制100、200、300、400、500、600 mg/L且pH均为5的2，5-DCBQ水溶液，各取1 mL分别移至已称好1 mg Tp-Azo的样品瓶中，超声分散后放置于摇床上30 min以达到吸附平衡。离心取上清液，稀释到合适倍数，过膜后使用紫外可见近红外光谱仪进行定量分析。

对实验数据进行Langmuir模型和Freundlich模型非线性拟合，Langmuir模型和Freundlich模型拟合公式分别见式（3）和式（4）：


$$\frac{C_{e}}{q_{e}}=\frac{1}{q_{m}K_{L}}+\frac{C_{e}}{q_{m}}$$ （3）

$$ln\;q_{e}=ln\;K_{F}+\frac{1}{n}ln\;C_{e}$$ （4）

其中，*q*_m_（mg/g）为最大吸附量，*C*_e_（mg/L）为平衡吸附浓度，*K*_L_（L/mg）为Langmuir模型常数，*K*_F_（（mg/g）/（L/mg）^1/^ *^n^* ）为Freundlich模型常数，*n*为Freundlich指数。

### 1.5 样品前处理

取实验室自来水和本地游泳池水，加入2，5-DCBQ配制成最终质量浓度为5 mg/L和50 mg/L的样品作为污染水样，调节pH为5，各取1 mL加入到1 mg Tp-Azo中，超声分散后放置于摇床上30 min以达到吸附平衡。离心取上清液，稀释合适倍数，过膜后使用紫外可见近红外光谱仪进行定量分析。2，5-DCBQ去除率（*R*，%）由式（5）进行计算：


$$R=\frac{\left(C_{blank}-C_{1}\right)}{C_{blank}}\times 100\%$$ （5）

其中，*C*_blank_（mg/L）为未经Tp-Azo吸附的样品测定的质量浓度；*C*_1_（mg/L）为经Tp-Azo吸附的样品测定的质量浓度。

## 2 结果与讨论

### 2.1 Tp-Azo的表征

2，5-DCBQ中两个氯原子（-Cl）和羰基（C=O）通过吸电子诱导效应，使得苯醌环整体成为强缺电子体系。在COF骨架中引入的富电子基团-N=N-，可显著提高整个COF骨架*π*电子云密度，从而通过电子给体-受体相互作用，增强偶氮COF对2，5-DCBQ的亲和力。基于此，我们选择含有-N=N-基团的非可逆*β*-酮烯胺连接的COF作为吸附剂。其制备过程如图1所示，选择Tp作醛基单体，Azo为氨基单体，通过互变异构反应，生成非可逆偶氮COF Tp-Azo。

**图1:**
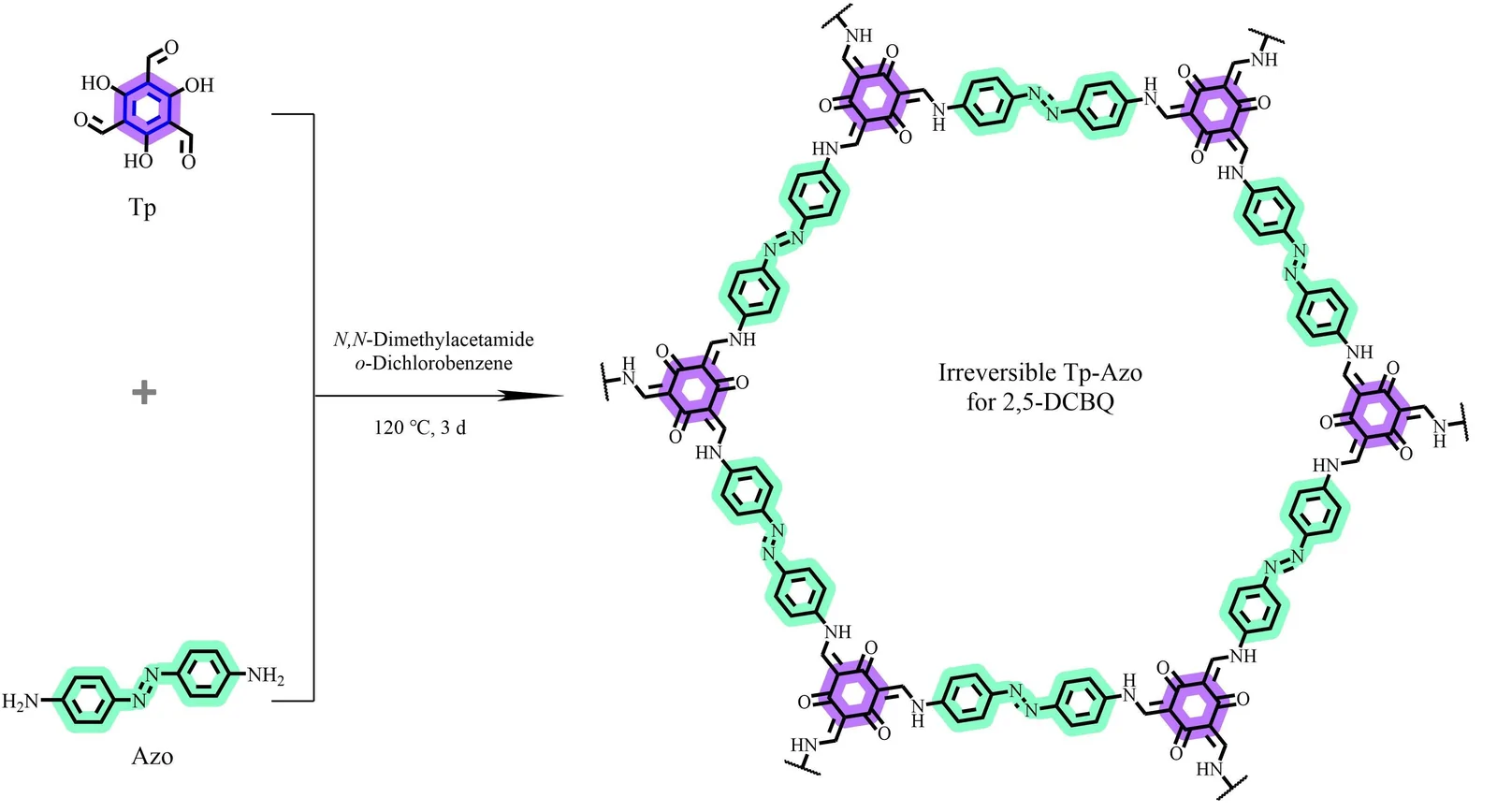
Tp-Azo的合成示意图

PXRD与FTIR分析共同证实了高结晶度Tp-Azo的成功合成。如图2a所示，所合成材料在3.2°处呈现一个尖锐且强烈的衍射峰，和软件精修的（Refined）理论衍射图谱高度匹配，加权全谱残差因子（*R*_wp_）和全谱残差因子（*R*_p_）均小于10%。此外，该衍射峰位置与文献［^18^］报道中相应材料的主衍射峰完全一致，证明了所制备Tp-Azo具有高度有序的结晶结构。Tp单体中-CHO的羰基伸缩振动峰（1 634 cm^‒1^）以及Azo单体中-NH_2_的N-H伸缩振动宽峰（3 200~3 500 cm^‒1^）在产物谱图中完全消失。与此同时，在Tp-Azo谱图中位于1 571 cm^‒1^处的强峰归属于芳香环C=C伸缩振动，而1 617 cm^‒1^处的尖锐峰则明确归属于新生成的-C=O的振动峰（图2b）。

**图2:**
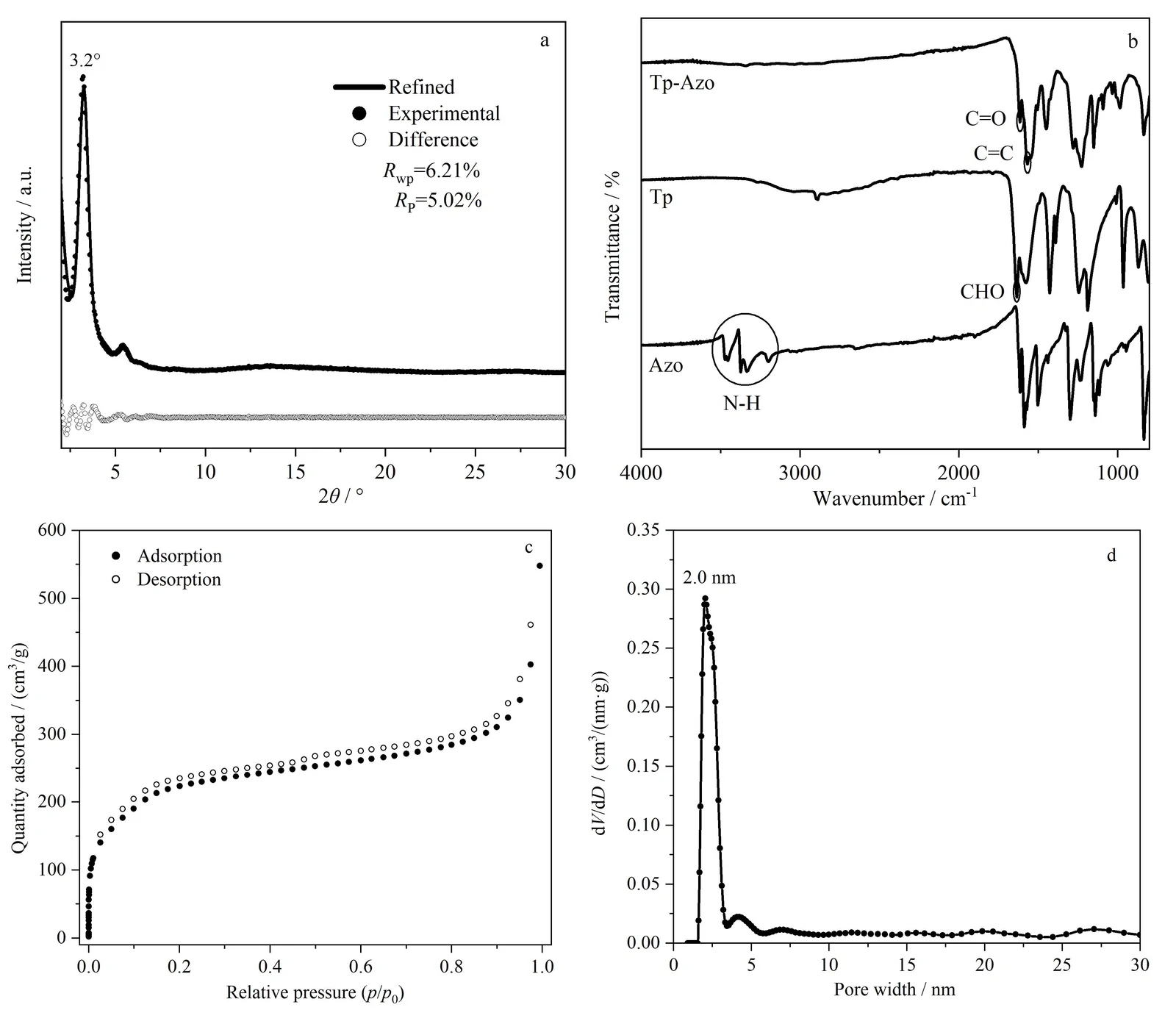
Tp-Azo的（a）PXRD衍射图、（b）红外光谱图、（c）N_2_吸附-脱附等温曲线和（d）孔径分布图

N_2_吸附-脱附曲线（图2c）表明了所制备的Tp-Azo材料具有介孔结构。通过BET模型计算，其比表面积为824.5 m^2^/g，表明材料可为吸附过程提供丰富的表面活性位点。基于DFT模型的孔径分布图（图2d）显示，其主要孔径集中在2.0 nm。这一孔径尺寸远大于目标污染物2，5-DCBQ的分子动力学直径（约0.8 nm）。这种“宽通道”确保了2，5-DCBQ分子能够进入孔道内部，从而充分利用其高比表面积，为实现高效吸附奠定了理想的结构基础。

### 2.2 Tp-Azo吸附性能考察

#### 2.2.1 定量检测方法

2，5-DCBQ在272 nm处存在一个紫外吸收特征峰，这是因为2，5-DCBQ的母体结构是对苯醌，对苯醌本身具有环己二烯二酮（醌式）结构，其C=O与C=C键形成交叉共轭体系，电子离域有限，因此在约250 nm处出现一个典型的*π*→*π* ^*^强跃迁吸收带，当对苯醌的2位和5位引入-Cl后，由于-Cl有孤对电子，扩大了共轭范围，降低了*π*→*π* ^*^跃迁的能隙，从而导致吸收峰红移。因此可以通过监控272 nm处的紫外吸收来对2，5-DCBQ进行定量。

为了保证数据的真实性和可靠性，我们选择了272 nm处吸收值小于2.0的数据对应的浓度绘制标准曲线。从不同浓度2，5-DCBQ水溶液的UV-Vis吸收光谱（图3a）和272 nm处的吸光度值与2，5-DCBQ浓度的关系图（图3b）可以看出，随着2，5-DCBQ浓度的增加，272 nm处的吸光度值呈现不断增加的趋势，在0.125~25 mg/L范围内，吸光度值线性增加，*R* ^2^为0.999 9。因此标准曲线完全可以用来定量分析2，5-DCBQ。在后续的实验中，样品经过离心、稀释、过膜，使用紫外可见近红外光谱仪进行定量分析。

**图3:**
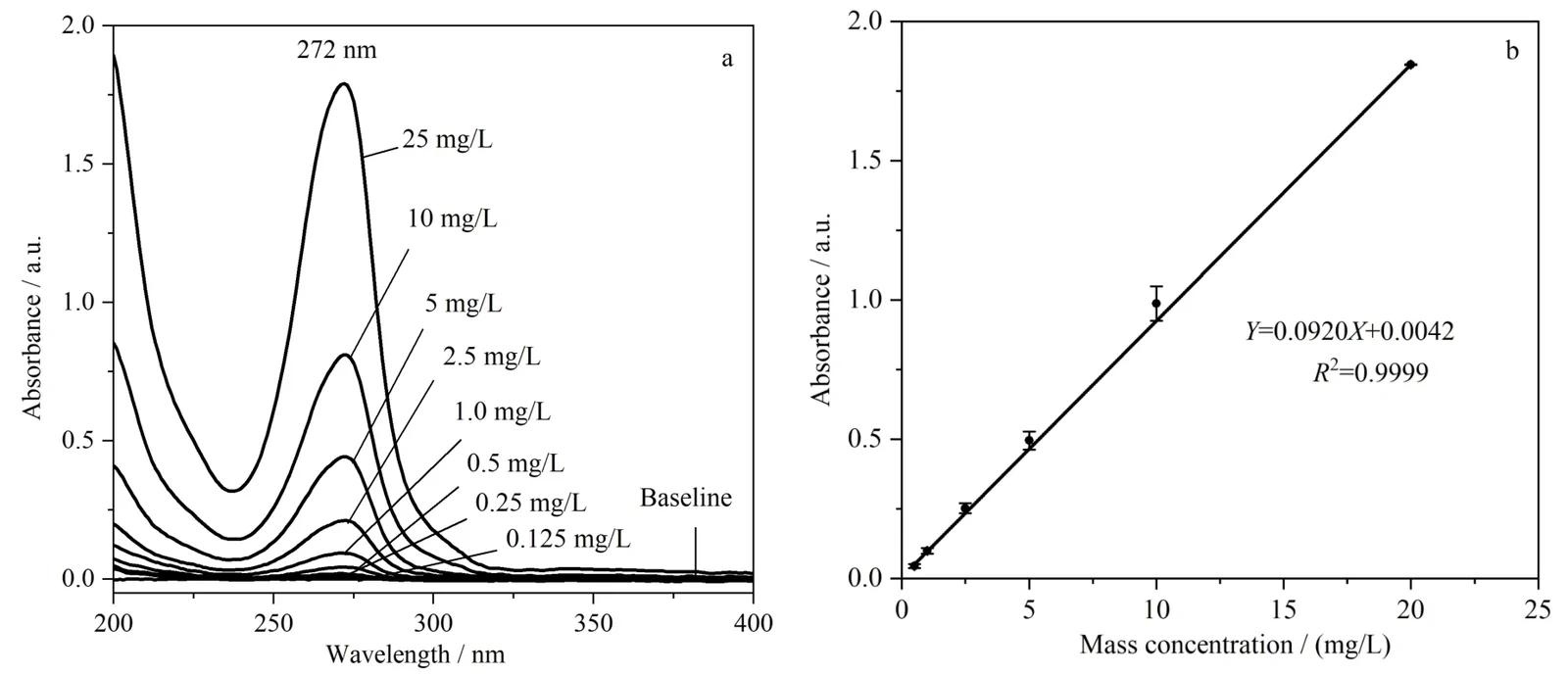
（a）不同浓度2，5-DCBQ水溶液的UV-Vis吸收光谱图；（b）272 nm处的吸光度值与2，5-DCBQ浓度的关系图

#### 2.2.2 pH对吸附性能的影响

2，5-DCBQ主要存在酸性条件下，这是因为在酸性环境中，氯主要以次氯酸形式存在，能更有效地将酚类等前体物氯化并氧化生成2，5-DCBQ。同时，酸性条件能抑制2，5-DCBQ的水解，使其在水中稳定存在。因此，本研究测试了pH 2~6范围内Tp-Azo对2，5-DCBQ的吸附性能（图4）。实验结果表明，在pH 2~3条件下，Tp-Azo易发生质子化，导致其疏水性减弱，同时高浓度的H^+^会屏蔽Tp-Azo与2，5-DCBQ之间的静电相互作用，从而降低其吸附效率。而在pH 4~6范围内，Tp-Azo对2，5-DCBQ均表现出较高的去除率。基于此，后续吸附实验选定在pH=5的条件下进行。

**图4:**
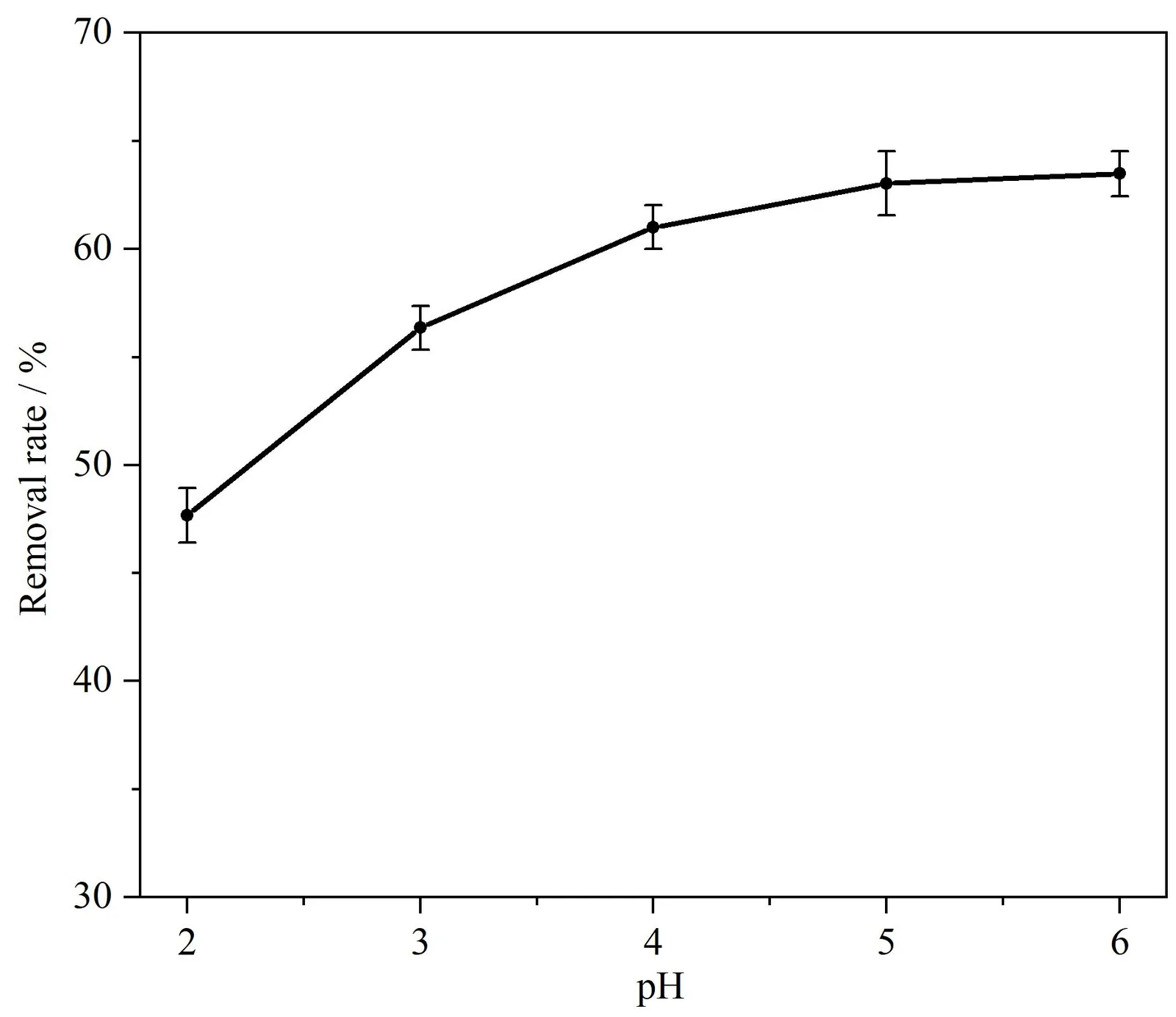
2，5-DCBQ 水溶液的pH对Tp-Azo吸附性能的影响（*n*=3）

#### 2.2.3 动力学吸附性能

动力学曲线（图5a）表明，Tp-Azo对2，5-DCBQ的吸附在3 min内迅速接近平衡，并于20 min内完全达到吸附平衡。以时间*t*为横轴，ln （*q*_e_ *-q_t_* ）为纵轴作图，对得到的散点进行伪一级动力学模型的线性拟合；以时间*t*为横轴，*t/q_t_* 作纵轴作图，对得到的散点进行伪二级动力学模型的线性拟合（图5b、5c）。结果显示，伪二级动力学模型的拟合度（*R* ^2^=0.998 7）远优于伪一级动力学模型（*R* ^2^=0.457 6）。这表明Tp-Azo对2，5-DCBQ的吸附过程主要受化学作用控制，而非物理传质过程。

**图5:**
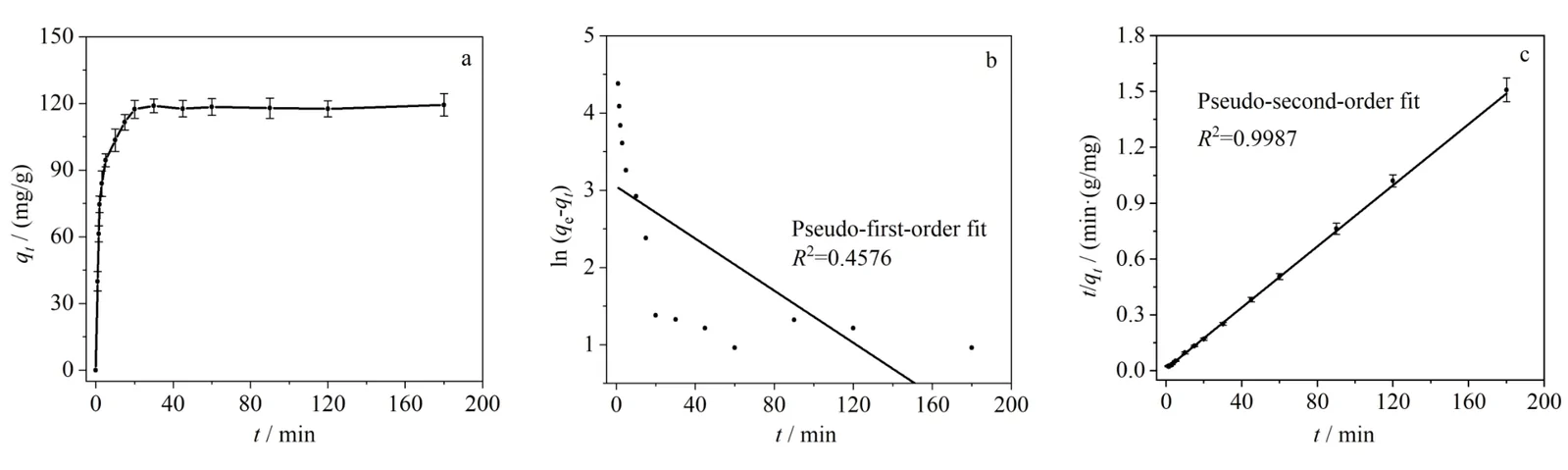
（a）Tp-Azo对2，5-DCBQ的吸附动力学曲线（0~180 min）；Tp-Azo吸附2，5-DCBQ的（b）伪一级和（c）伪二级动力学线性拟合图

#### 2.2.4 等温吸附性能

吸附等温线结果表明，Tp-Azo对2，5-DCBQ的平衡吸附量随其初始浓度的增加而增大。分别采用Langmuir和Freundlich模型对实验数据进行非线性拟合（图6），结果显示Langmuir模型的拟合相关系数（*R* ^2^=0.992 1）高于Freundlich模型（*R* ^2^=0.915 1），表明该吸附过程更符合Langmuir模型，吸附行为以单分子层吸附为主。进一步，计算出Tp-Azo对2，5-DCBQ的最大吸附量*q*_m_为427.4 mg/g，与其他报道的DBPs去除方法相比（表1），Tp-Azo对2，5-DCBQ的吸附平衡时间短，吸附容量高。本文制备的高比表面积Tp-Azo含有大量富电子的-N=N-基团，能与缺电子的2，5-DCBQ形成电子给体-受体相互作用。此外，Tp-Azo骨架以芳香环为主，能和2，5-DCBQ形成*π-π*相互作用。疏水的Tp-Azo还可以通过疏水相互作用吸附2，5-DCBQ。上述多种作用协同，共同促进了Tp-Azo对2，5-DCBQ的高效吸附。

**图6:**
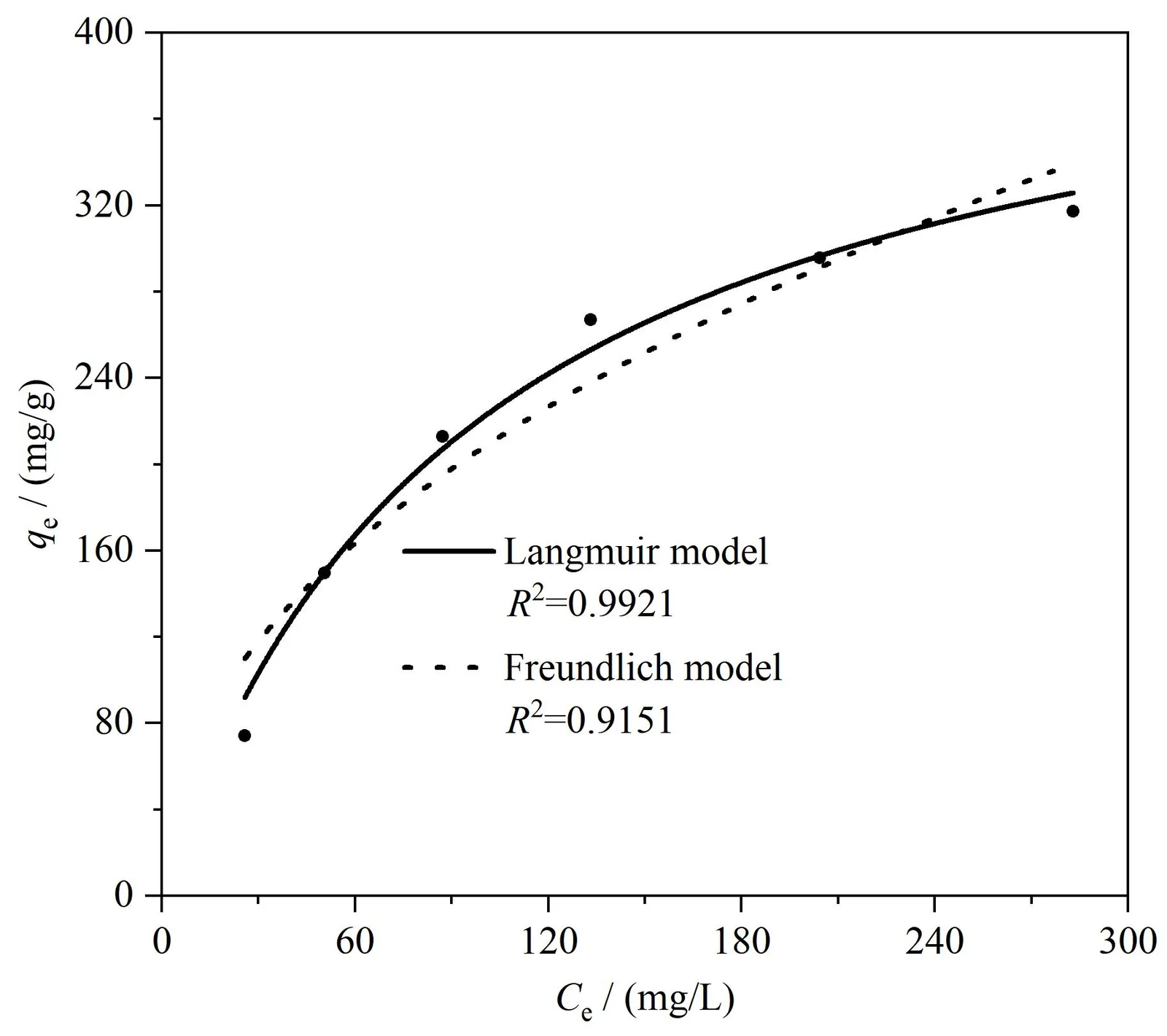
Tp-Azo吸附2，5-DCBQ的Langmuir模型和Freundlich模型的非线性拟合

**表1:** 不同吸附剂对DBPs吸附性能的对比

Adsorbent	Contaminants	*q*_m_	Equilibrium time/min	Ref.
Powdered activated carbon	trihalomethanes and haloacetonitriles	78.5-128.1 μg/L	160	［^19^］
Fe_3_O_4_@SiO_2_@PDA@NH_2_-MIL-53 （Fe）	2，6-DCBQ	109.3 mg/g	60	［^20^］
Graphene oxide	trichloromethane	450 μg/g	50	［^21^］
Polymer brush-grafted cotton fiber	aromatic halogenated DBPs	14.8-89.5 mg/g	6	［^22^］
MOF （UiO-66-NMe^3+^）	2，4-dichloro-phenoxyacetic acid	279 mg/g	250	［^23^］
COF Tp-Azo	2，5-DCBQ	427.4 mg/g	20	this work

### 2.3 实际样品分析

为了评估Tp-Azo在真实水样中2，5-DCBQ的吸附性能，本研究选取某游泳池水和实验室自来水为研究对象，上述样品经0.22 μm水系膜过滤后，未能直接检出2，5-DCBQ。分别向游泳池水和实验室自来水样品中加标2，5-DCBQ至5 mg/L和50 mg/L以模拟污染水样，使用Tp-Azo对加标后的水样进行吸附去除。吸附实验结果表明，在5 mg/L水平下，Tp-Azo对游泳池水和实验室自来水中2，5-DCBQ的去除率分别为96.2%和96.6%，在50 mg/L的高浓度下，Tp-Azo对两种水样中2，5-DCBQ的去除率分别为87.9%和88.7%，表明Tp-Azo具备优异的2，5-DCBQ去除能力（图7）。实际水样中的卤代消毒副产物的污染水平大多为μg/L级^［1，24，25］^，使用Tp-Azo处理，有望实现2，5-DCBQ的高效去除。

**图7:**
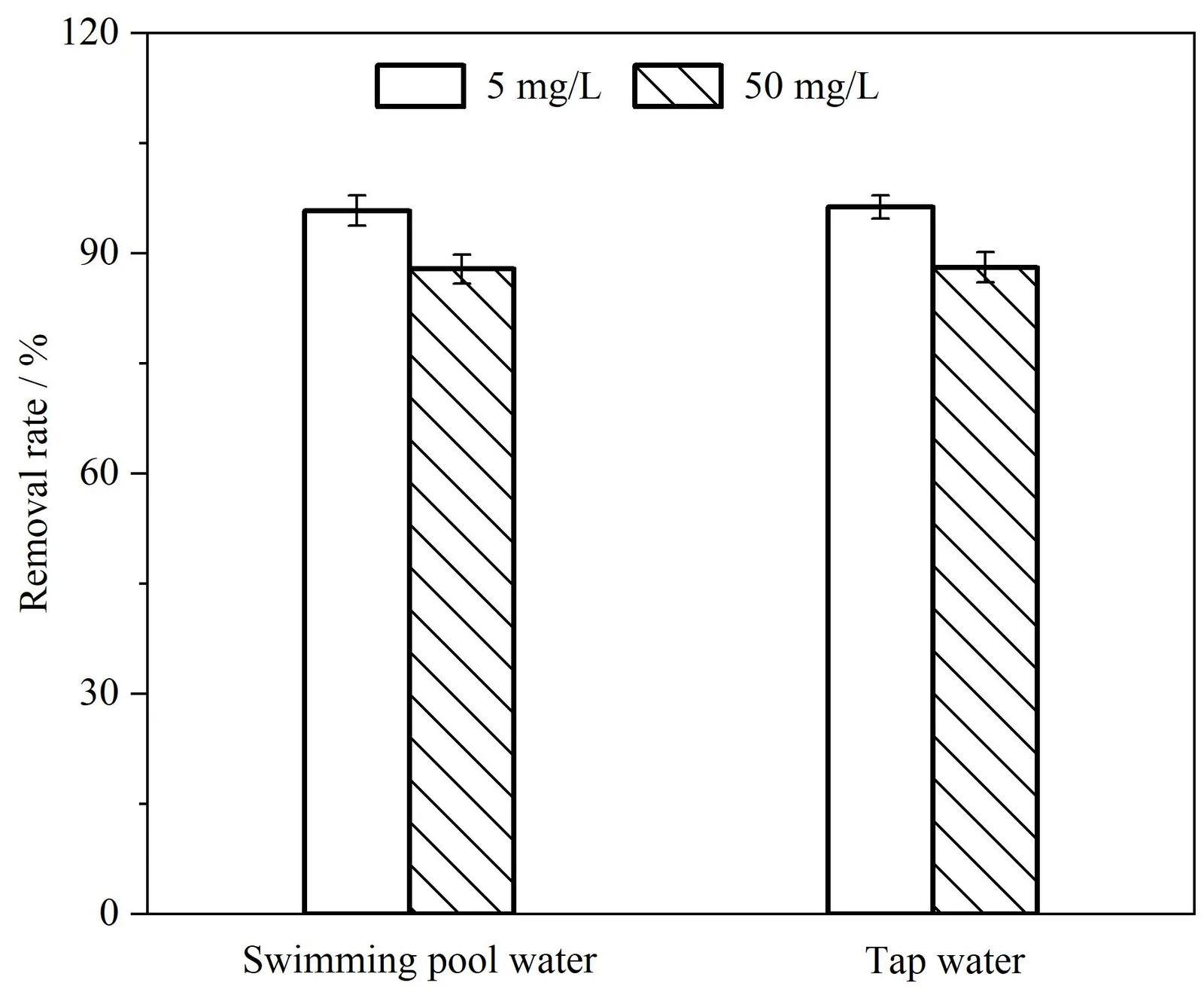
实际水样中2，5-DCBQ的去除率（*n*=3）

## 3 结论

本研究通过互变异构反应制备了一种非可逆的酮烯胺式偶氮苯COF Tp-Azo用于典型卤代苯醌2，5-DCBQ的去除。在电子给体-受体相互作用、*π-π*相互作用和疏水相互作用等多重作用下，Tp-Azo对实际水样中2，5-DCBQ实现了高效去除。与已报道的用于去除DBPs的工作相比，基于Tp-Azo的2，5-DCBQ去除表现出更高的吸附容量以及更短的平衡时间。该工作不仅为卤代DBPs的去除提供了新材料和新思路，更为环境水中DBPs的治理提供了高效的解决方案。
